# Childhood cancer and residential proximity to petrol stations: a nationwide registry-based case–control study in Switzerland and an updated meta-analysis

**DOI:** 10.1007/s00420-021-01767-y

**Published:** 2021-10-15

**Authors:** Antonella Mazzei, Garyfallos Konstantinoudis, Christian Kreis, Manuel Diezi, Roland A. Ammann, Marcel Zwahlen, Claudia Kühni, Ben D. Spycher

**Affiliations:** 1 Institute of Social and Preventive Medicine (ISPM), University of Bern, Mittelstrasse 43, 3012 Bern, Switzerland; 2 MRC Centre for Environment and Health, Department of Epidemiology and Biostatistics, School of Public Health, Imperial College London, London, UK; 3 Centre Hospitalier Universitaire Vaudois, Lausanne University Hospital, Lausanne, Switzerland; 4 Pediatric Hematology/Oncology, Department of Pediatrics, Inselspital, Bern University Hospital, University of Bern, Bern, Switzerland; 5 Kinderaerzte, KurWerk, Burgdorf, Switzerland

**Keywords:** Hematological cancer, Air pollution, Solvents, Benzene

## Abstract

**Purpose:**

Benzene is a known carcinogen for adult leukemia. Exposure to benzene through parental occupation and the use of household products has been associated with childhood leukemia (CL). Ambient benzene has also been associated with CL and central nervous system (CNS) tumors. We aimed to investigate whether the higher ambient levels of benzene in proximity of petrol stations are associated with a greater risk of childhood cancers, leukemia, and CNS tumors.

**Methods:**

We identified children diagnosed with cancer at age 0–15 years during 1985–2015 from the Swiss Childhood Cancer Registry and selected 10 age and sex-matched controls per case from national censuses. We calculated the distance from children’s home to the nearest petrol station using precise geocodes. We estimated odds ratios using conditional logistic regression adjusting for ambient levels of NO_2_, distance to highways, level of urbanization, and presence of a cantonal cancer registry. In addition, we ran a meta-analysis pooling current results for CL with those of previous studies.

**Results:**

We identified 6129 cases, of which 1880 were leukemias and 1290 CNS tumors. 24 cases lived within 50 m from a petrol station. The adjusted odds ratio of a cancer diagnosis for children thus exposed compared to unexposed children (> 500 m) was 1.29 (0.84–1.98) for all cancers combined, 1.08 (0.46–2.51) for leukemia, and 1.30 (0.51–3.35) for CNS tumors. During 2000–2015, when exposure assessment was more precise, the adjusted odds ratio for any cancer diagnosis was 1.77 (1.05–2.98). The summary relative risk estimate for CL in the meta-analysis including four studies was 2.01 (1.25–3.22).

**Conclusions:**

Our study provides weak support for an increased risk of childhood cancers among children living close to petrol stations. A meta-analysis including our study suggests an increased risk for CL.

**Supplementary Information:**

The online version contains supplementary material available at 10.1007/s00420-021-01767-y.

## Introduction

Little is known about potential environmental risk factors of cancers in childhood. The most common cancers affecting children in most world regions are leukemias and tumors of the central nervous system (CNS) (Steliarova-Foucher et al. 2017). Benzene is a designated Group I carcinogen by the International Agency for Research on Cancer (IARC) and an established risk factor for acute myeloid leukemia in adults, with positive associations also being noted for chronic myeloid leukemia and the broad category of non-Hodgkin lymphoma (IARC 2017). There is also evidence of a link with childhood leukemia through parental occupational exposure and the home use of products containing benzene as solvent (Carlos-Wallace et al. 2016). Benzene is also present at lower concentrations in outdoor air from industrial emissions or motor vehicle exhausts (Wilbur et al. 2007). There is growing evidence of an association between ambient levels of benzene at residence, especially as a component of traffic-related air pollution, and childhood leukemia, particularly acute myeloid leukemia (Filippini et al. 2019; Raaschou-Nielsen et al. 2018). Results from studies of occupational exposure to benzene and adult CNS tumors are inconsistent (IARC 2017). For childhood CNS tumors, several studies found a positive association with parental occupational exposure (Keegan et al. 2013; Peters et al. 2014), whereas the few studies on ambient exposure reported elevated risks only for some subtypes but not for others (Danysh et al. 2015; Raaschou-Nielsen et al. 2018; von Ehrenstein et al. 2016).

In Switzerland, approximately 75% of benzene emissions originate from the production, distribution, and burning of fuel (i.e. from refineries, petrol stations, and motor vehicle combustion engines) (SAEFL 2004). Benzene concentration levels in outdoor air have been falling since the 1980s. This was the result first of strict regulation and later a ban of its use as a solvent and of new regulation about fuel quality enacted by the European Union and implemented in Switzerland stipulating a maximum benzene content in petrol of 1% volume as of January 2000. Petrol stations contribute significantly to the ambient benzene concentration in their immediate environment (Karakitsios et al. 2007) but it remains unclear if children who live nearby and are thus exposed to higher concentrations incur an excess risk. Concentrations are highest near the petrol pumps but have also been found to exceed 5 µg/m^3^, the maximum allowable ambient level in the EU, at the adjacent roadside (Karakitsios et al. 2007; Morales Terrés et al. 2010). Wider dispersion depends on the built environment surrounding the site and the weather, but ambient concentrations revert to background levels at around 50 m (Morales Terrés et al. 2010). A report by the Bavarian Ministry for the Environment estimated that the annual mean indoor air concentration of benzene was 1.0 μg/m^3^ and 0.4 μg/m^3^ higher in residences located at 8–12 and 10–40 m away from a petrol station, respectively, compared to residences further away (Bayerisches Landesamt für Umwelt 2013).

Three case–control studies investigated the risk of childhood leukemia associated with residential proximity to petrol stations (Brosselin et al. 2009; Harrison et al. 1999; Steffen et al. 2004). All three reported positive associations but effect estimates varied substantially. The two French studies (Brosselin et al. 2009; Steffen et al. 2004) considered children exposed if their place of residence was directly adjacent to a petrol station at any time between conception and diagnosis (or time of interview for controls). Exposure was assessed based on interviews with children’s mothers and thus might be subject to recall bias. By contrast, the British study used georeferenced data to calculate actual distance defining as exposed children living within 100 m of a petrol station at diagnosis (Harrison et al. 1999). Two meta-analyses of these three studies suggest an increased risk of childhood leukemia among children living in close proximity to petrol stations (Carlos-Wallace et al. 2016; Steinmaus and Smith 2017), though with substantially differing estimates of summary relative risks due to differences in methodology. The evidence base thus consists of only few studies with widely differing effect estimates possibly due to small numbers of exposed cases and varying timing of exposure assessment.

In this nationwide case–control study, we aimed to investigate whether children living in close proximity to petrol stations (≤ 50 m) are at greater risk of developing childhood cancer. We separately investigated all diagnostic groups combined, leukemia, and CNS tumors using exposure at birth and at diagnosis. We also considered possible differences in associations by sub-periods accounting for the decline in background levels of benzene over the 1985–2015 study period.

## Methods

### Cases

We identified all cases of childhood cancer diagnosed between 1985 and 2015 in children and adolescents aged 0–15 years living in Switzerland at diagnosis from the Swiss Childhood Cancer Registry (SCCR). The SCCR is a nationwide cancer registry with an estimated completeness of over 90% since 1985 and about 95% since the mid-1990s (Schindler et al. 2015). In the SCCR, cases are classified according to the International Classification of Childhood Cancer, Third Edition (ICCC3) (Steliarova-Foucher et al. 2005). Addresses of cases at birth and diagnosis were extracted from the SCCR and geocoded electronically using the georeferenced postal addresses registry provided by the Swiss postal service (GeoPost Coordinates) or manually using the geoportal maintained by the Federal Office of Topography (swisstopo) (http://map.geo.admin.ch). We obtained precise geocodes to within 50 m for approximately 93% of the available cases (Konstantinoudis et al. 2017).

### Outcomes

We conducted the analysis for the following outcomes: all cancers (ICCC3 diagnostic groups I.-XII. and Langerhans cell histiocytosis), leukemia (ICCC3 main group I.), and CNS tumors (III.). We selected these outcomes a priori based on previous studies and the number of cases in the SCCR available for analysis (minimum threshold 1000 cases). For leukemia, we conducted a separate analysis for cases diagnosed under 5 years of age.

### Controls

We obtained individual data on the Swiss resident population, including place of residence, from the Swiss national censuses (conducted in 1990, 2000, and annually from 2010 onward) through the Swiss National Cohort. We randomly sampled ten controls per case matched by age, sex, and census corresponding to year of diagnosis of the index cases. For cases diagnosed between census years, we sampled controls probabilistically from either the previous or following census, attributing more weight to the census closest to the index cases’ year of diagnosis. For example, if a case was diagnosed in 1993, we sampled controls with probability 0.7 and 0.3 from the 1990 and 2000 census, respectively. We followed the same approach to select controls for analysis at time of birth, but only sampled children aged < 1 year old at time of census. We selected ten controls per case to approach statistical power (Taylor 1986).

### Exposure

We extracted exact geocodes of all petrol stations registered in the Swiss Business Censuses, which have been conducted by the Swiss Federal Office of Statistics in 1995, 1998, 2001, 2005, 2008, and annually since 2011. We calculated the minimum (Euclidean) distance of the cases’ and controls’ residence to the nearest petrol station recorded in the latest business census preceding the index cases’ diagnosis, except for the years 1985–1994, for which we used the 1995 Swiss Business Census for lack of an earlier census. We only considered the locations of petrol stations, excluding automotive repair garages or service stations. We calculated distance to petrol stations based on the locations recorded in a single business census year for each case and its matched controls, even for cases diagnosed between population census years, for whom we sampled controls randomly from both the preceding and the following census.

### Potential confounders

We considered confounding by the following factors: (1) outdoor air concentration level of NO_2_ (µg/m^3^) as estimated by the national dispersion model PolluMap in 1990, 2000, and 2010 for a nationwide grid of cells of 200 × 200 m (Heldstab et al. 2011); (2) traffic-related air pollution measured as the distance to the nearest highway (0– < 100, 100– < 250, 250– < 500, ≥ 500 m); (3) Swiss index of socio-economic position (SEP) of the immediate neighborhood area (range 0–100) (Panczak et al. 2012); and (4) degree of urbanization of the municipality of residence (urban, peri-urban, and rural) as defined by the Swiss Federal Office of Statistics. The rationale for including these confounders was to adjust for differences in background levels of air pollution, which vary between rural and urban areas. Swiss SEP was included to adjust for any influence of socio-economic factors. In addition, we adjusted for (5) years of existence of a general cantonal cancer registry to account for potential differences in registration coverage (Konstantinoudis et al. 2020). While the SCCR has nationwide coverage, general cantonal registries were consulted, where they existed, to identify any missed cases.

### Statistical analysis

We used conditional logistic regression conditioning on matched case–control sets to estimate odds ratios (ORs) and 95% confidence intervals for the following categories of distance to petrol stations: ≤ 50, > 50–100, > 100–250, > 250–500, > 500 m (reference category). No cases nor controls were lost to the analyses due to missing data except for missing geocodes. We report ORs unadjusted and adjusted for the potential confounders considered. We carried out separate analyses by restricting the study period to the more recent periods 1995–2015 and 2000–2015, respectively. The rationale for these analyses was: first, to reduce the risk of exposure misclassification due to the lack of contemporary data on the locations of petrol stations before 1995; and, second, to allow for potentially altered exposure-outcome relationships after the enforcement of tighter clean air regulations, in particular limiting the benzene content of petrol to 1% volume in the year 2000.

In an additional meta-analysis, we pooled our results with those of the three previous studies included by Steinmaus and Smith (2017) to estimate the summary relative risk of childhood leukemia. We extracted odds ratios and 95% confidence intervals from the maximally adjusted logistic regression models and estimated the summary relative risk using a random-effects model with restricted maximum likelihood estimators.

We used the R language for statistical computing version 3.6.0 for all analyses, including GIS calculations to determine levels of exposure (R Core Team 2019).

## Results

### Study population

In total, 6151 cases of childhood cancer registered in the SCCR and diagnosed between 1985 and 2015 were eligible for analysis. After discarding 22 cases with missing geocodes, we included 6129 cases for all cancers combined in the analysis at diagnosis, 1880 of which were leukemias and 1290 CNS tumors. For the analysis at birth, after excluding 1222 cases born before 1985 or whose birthplace was outside of Switzerland or uncertain and discarding eight cases with missing geocodes at birth, we included 4921 cases for all cancers combined, 1542 of which were leukemias and 1046 CNS tumors.

The characteristics of the cases and controls are presented in Table 1. Most children residing in close proximity to a petrol station lived in urban areas (80.0%). Compared to the reference category (> 500 m), children in close proximity to a petrol station were also more likely to reside in a low socio-economic position (SEP) neighborhood (35.0% vs. 25.1%) and to be exposed to levels of outdoor air pollution above 30 µg/m^3^ NO_2_ (40.6% vs. 16.4%; Table 1).

**Table 1:** Characteristics of cases and controls by proximity to petrol stations at time of index cases’ diagnosis

	Cases										Controls									
	0–50 m		> 50–100 m		> 100–250 m		> 250–500 m		> 500 m		0–50 m		> 50–100 m		> 100–250 m		> 250–500 m		> 500 m	
	*n* = 24 (%)		*n* = 51 (%)		*n* = 296 (%)		*n* = 801 (%)		*n* = 4′957 (%)		*n* = 180 (%)		*n* = 520 (%)		*n* = 3′160 (%)		*n* = 7′337 (%)		*n* = 50′093 (%)	
Sex																				
Male	8	(33.3)	26	(51.0)	149	(50.3)	440	(54.9)	2′781	(56.1)	99	(55.0)	275	(52.9)	1′737	(55.0)	4′059	(55.3)	27′870	(55.6)
Female	16	(66.7)	25	(49.0)	147	(49.7)	361	(45.1)	2′176	(43.9)	81	(45.0)	245	(47.1)	1′423	(45.0)	3′278	(44.7)	22′223	(44.4)
Year of Birth																				
1970–1979	3	(12.5)	3	(5.9)	21	(7.1)	52	(6.5)	359	(7.2)	20	(11.1)	29	(5.6)	212	(6.7)	501	(6.8)	4′081	(8.1)
1980–1989	6	(25.0)	14	(27.5)	75	(25.3)	190	(23.7)	1′296	(26.1)	39	(21.7)	133	(25.6)	774	(24.5)	1′751	(23.9)	14′812	(29.6)
1990–1999	2	(8.3)	18	(35.3)	97	(32.8)	291	(36.3)	1′735	(35.0)	66	(36.7)	144	(27.7)	1′013	(32.1)	2′255	(30.7)	16′056	(32.1)
2000–2009	12	(50.0)	12	(23.5)	76	(25.7)	201	(25.1)	1′298	(26.2)	43	(23.9)	158	(30.4)	855	(27.1)	2′100	(28.6)	11′737	(23.4)
2010–2015	1	(4.2)	4	(7.8)	26	(8.8)	66	(8.2)	263	(5.3)	12	(6.7)	56	(10.8)	306	(9.7)	730	(9.9)	3′407	(6.8)
N-Miss	0	(0.0)	0	(0.0)	1	(0.3)	1	(0.1)	6	(0.1)	0	(0.0)	0	(0.0)	0	(0.0)	0	(0.0)	0	(0.0)
Degree of urbanization																				
Urban	20	(83.3)	43	(84.3)	262	(88.5)	688	(85.9)	2′669	(53.8)	144	(80.0)	435	(83.7)	2′751	(87.1)	6′448	(87.9)	26′091	(52.1)
Peri-urban	3	(12.5)	5	(9.8)	15	(5.1)	73	(9.1)	1′341	(27.1)	17	(9.4)	54	(10.4)	228	(7.2)	532	(7.3)	13′738	(27.4)
Rural	1	(4.2)	3	(5.9)	19	(6.4)	40	(5.0)	947	(19.1)	19	(10.6)	31	(6.0)	181	(5.7)	357	(4.9)	10′264	(20.5)
Swiss-SEP index																				
1st quintile (low SEP)	12	(50.0)	18	(35.3)	94	(31.8)	231	(28.8)	1′213	(24.5)	63	(35.0)	206	(39.6)	1′090	(34.5)	2′054	(28.0)	12′592	(25.1)
2nd quintile	4	(16.7)	16	(31.4)	68	(23.0)	156	(19.5)	986	(19.9)	48	(26.7)	128	(24.6)	653	(20.7)	1′467	(20.0)	10′414	(20.8)
3rd quintile	5	(20.8)	5	(9.8)	49	(16.6)	151	(18.9)	991	(20.0)	37	(20.6)	75	(14.4)	559	(17.7)	1′337	(18.2)	9′538	(19.0)
4th quintile	1	(4.2)	7	(13.7)	58	(19.6)	151	(18.9)	930	(18.8)	21	(11.7)	67	(12.9)	535	(16.9)	1′349	(18.4)	9′348	(18.7)
5th quintile (high SEP)	2	(8.3)	5	(9.8)	27	(9.1)	112	(14.0)	837	(16.9)	11	(6.1)	44	(8.5)	323	(10.2)	1′130	(15.4)	8′201	(16.4)
Distance to highway (m)																				
0–100	1	(4.2)	5	(9.8)	8	(2.7)	23	(2.9)	93	(1.9)	9	(5.0)	18	(3.5)	89	(2.8)	183	(2.5)	727	(1.5)
100–250	1	(4.2)	0	(0.0)	15	(5.1)	51	(6.4)	197	(4.0)	14	(7.8)	35	(6.7)	213	(6.7)	488	(6.7)	2′032	(4.1)
250–500	2	(8.3)	6	(11.8)	56	(18.9)	121	(15.1)	405	(8.2)	23	(12.8)	73	(14.0)	435	(13.8)	1′006	(13.7)	4′122	(8.2)
> 500	20	(83.3)	40	(78.4)	217	(73.3)	606	(75.7)	4′262	(86.0)	134	(74.4)	394	(75.8)	2′423	(76.7)	5′660	(77.1)	43′212	(86.3)
NO_2_ (µg/m^3^)^1^																				
(0,20]	3	(12.5)	6	(11.8)	31	(10.5)	110	(13.7)	2′158	(43.5)	21	(11.7)	59	(11.3)	396	(12.5)	968	(13.2)	22′204	(44.3)
(20,30]	15	(62.5)	27	(52.9)	131	(44.3)	393	(49.1)	1′912	(38.6)	86	(47.8)	269	(51.7)	1′561	(49.4)	3′681	(50.2)	19′652	(39.2)
(30,40]	4	(16.7)	13	(25.5)	94	(31.8)	206	(25.7)	666	(13.4)	51	(28.3)	133	(25.6)	837	(26.5)	1′927	(26.3)	6′382	(12.7)
(40,50]	1	(4.2)	1	(2.0)	26	(8.8)	63	(7.9)	163	(3.3)	12	(6.7)	43	(8.3)	237	(7.5)	568	(7.7)	1′401	(2.8)
(50,80]	1	(4.2)	4	(7.8)	14	(4.7)	29	(3.6)	58	(1.2)	10	(5.6)	16	(3.1)	129	(4.1)	193	(2.6)	454	(0.9)
Years of cantonal registry^2^																				
[0,10]	2	(8.3)	18	(35.3)	68	(23.0)	212	(26.5)	2′137	(43.1)	48	(26.7)	142	(27.3)	921	(29.1)	2′175	(29.6)	23′054	(46.0)
(10,20]	8	(33.3)	10	(19.6)	29	(9.8)	52	(6.5)	133	(2.7)	13	(7.2)	48	(9.2)	255	(8.1)	518	(7.1)	1′491	(3.0)
(20,30]	14	(58.3)	23	(45.1)	199	(67.2)	537	(67.0)	2′687	(54.2)	119	(66.1)	330	(63.5)	1′984	(62.8)	4′644	(63.3)	25′548	(51.0)
Period of time																				
1985–2000	7	(29.2)	24	(47.1)	131	(44.3)	356	(44.4)	2′394	(48.3)	95	(52.8)	212	(40.8)	1′297	(41.0)	3′040	(41.4)	24′476	(48.9)
2000–2015	17	(70.8)	27	(52.9)	165	(55.7)	445	(55.6)	2′563	(51.7)	85	(47.2)	308	(59.2)	1′863	(59.0)	4′297	(58.6)	25′617	(51.1)

^1^Estimated outdoor air concentration level of NO_2_ at children’s place of residence

^2^Number of years of existence of a general cantonal cancer registry

In Switzerland, the number of petrol stations grew by 37% from 828 in 1995, the year of the first Swiss Business Census, to 1136 in 2015 (Figure S1 in the online supplementary material). Distances from cases’ home to the closest petrol station ranged from 0 m (i.e. children living in a building with a built-in petrol station) to 28.4 km. 24 cancer cases lived in close proximity to a petrol station (≤ 50 m) at diagnosis, six of which were diagnosed with leukemia and five with CNS tumors (Table 2).

**Table 2:** Associations between childhood cancer and proximity to petrol stations at diagnosis

Outcome	Distance	Cases	OR^a^	95% CI^b^	Adj OR^c^	95% CI^b^
All cancers	0–50 m	24	1.35	(0.88–2.07)	1.29	(0.84–1.98)
	> 50–100 m	51	0.99	(0.74–1.32)	0.95	(0.71–1.27)
	> 100–250 m	296	0.95	(0.84–1.07)	0.91	(0.80–1.03)
	> 250–500 m	801	1.10	(1.02–1.19)	1.06	(0.98–1.15)
	> 500 m	4′957	1.00		1.00	
Leukemia	0–50 m	6	1.10	(0.47–2.56)	1.08	(0.46–2.51)
	> 50–100 m	15	0.88	(0.52–1.49)	0.86	(0.50–1.46)
	> 100–250 m	100	1.03	(0.83–1.28)	1.01	(0.81–1.25)
	> 250–500 m	240	1.07	(0.93–1.24)	1.05	(0.90–1.22)
	> 500 m	1′519	1.00		1.00	
Leukemia, 0–5 y	0–50 m	3	0.91	(0.28–2.99)	0.88	(0.27–2.87)
	> 50–100 m	9	0.87	(0.44–1.73)	0.82	(0.41–1.64)
	> 100–250 m	51	1.00	(0.75–1.35)	0.95	(0.70–1.29)
	> 250–500 m	121	1.05	(0.86–1.29)	1.01	(0.82–1.25)
	> 500 m	771	1.00		1.00	
CNS tumors	0–50 m	5	1.47	(0.57–3.75)	1.30	(0.51–3.35)
	> 50–100 m	10	0.99	(0.51–1.90)	0.92	(0.48–1.78)
	> 100–250 m	63	0.93	(0.71–1.21)	0.89	(0.68–1.17)
	> 250–500 m	155	1.01	(0.85–1.21)	0.97	(0.80–1.17)
	> 500 m	1′057	1.00		1.00	

^a^Odds ratio of unadjusted conditional logistic regression model

^b^95% confidence interval

^c^Odds ratio of conditional logistic regression model adjusted for ambient level of NO_2_, distance to the nearest highway, socio-economic position of the immediate neighborhood area, degree of urbanization of the municipality of residence, and years of existence of a general cantonal cancer registry

### Exposure at diagnosis

**Fig. 1:**
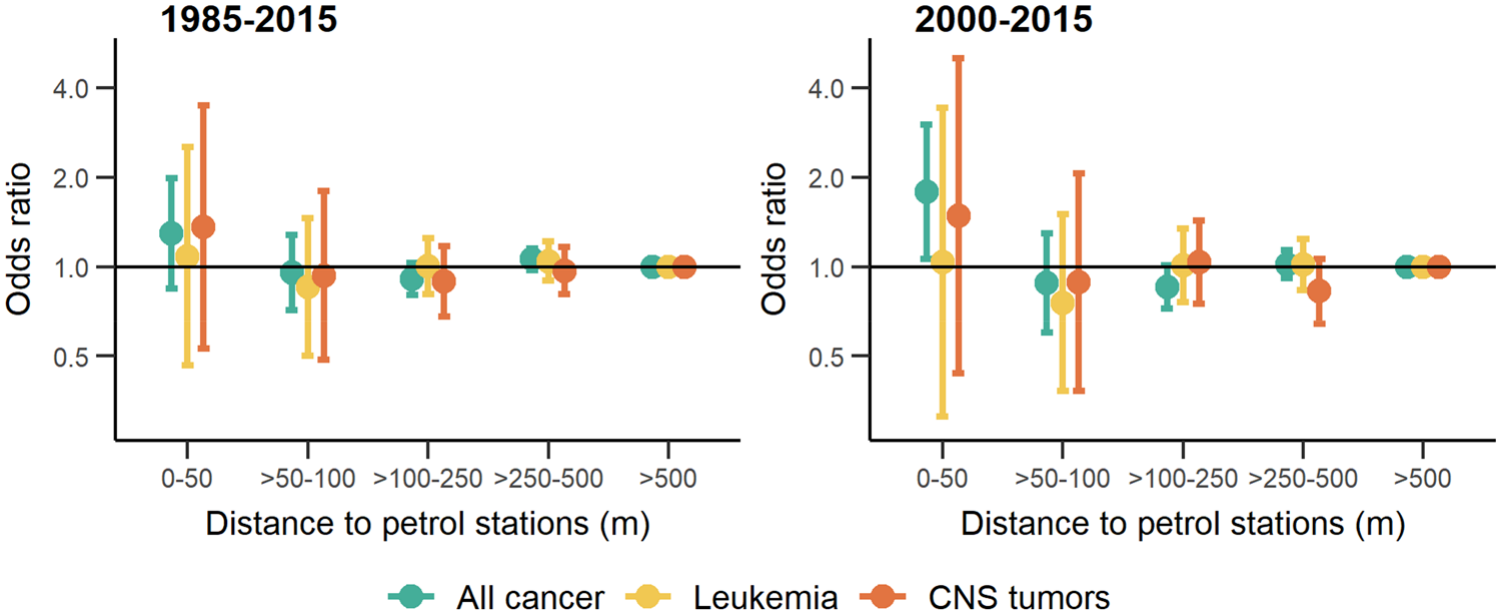
Associations between childhood cancer and proximity to petrol stations at diagnosis during 1985–2015 (left panel) and during 2000–2015 (right panel). Odds ratios and 95%-confidence intervals of conditional logistic regression models by distance category for different diagnostic groups adjusted for ambient level of NO_2_, distance to the nearest highway, socio-economic position of the immediate neighborhood area, degree of urbanization of the municipality of residence, and years of existence of a general cantonal cancer registry

The unadjusted odds ratio of a cancer diagnosis for children living in close proximity to a petrol station compared to unexposed children (> 500 m) was 1.35 (95%-confidence interval: 0.88–2.07) for all cancers combined, 1.10 (0.47–2.56) for leukemia, 0.91 (0.28–2.99) for leukemia in children under 5 years of age, and 1.47 (0.57–3.75) for CNS tumors (Table 2). Adjusting the analyses for confounding did not materially alter the results (Table 2 and Fig. 1). Effect estimates for any cancer were higher when we restricted to more recent time periods: the adjusted odds ratio of any cancer diagnosis was 1.41 (0.86–2.32) and 1.77 (1.05–2.98) during 1995–2015 (Table S1) and 2000–2015 (Table 3 and Fig. 1), respectively. Some variation was also observed in the odds ratios for leukemia and CNS tumors in more recent time periods but confidence intervals were wide due to the small number of exposed cases (Tables 3 and S1).

**Table 3:** Associations between childhood cancer and proximity to petrol stations at diagnosis, 2000–2015

Outcome	Distance	Cases	OR^a^	95% CI^b^	Adj OR^c^	95% CI^b^
All cancers	0–50 m	17	1.82	(1.09–3.06)	1.77	(1.05–2.98)
	> 50–100 m	29	0.90	(0.61–1.32)	0.88	(0.60–1.29)
	> 100–250 m	169	0.87	(0.74–1.02)	0.86	(0.72–1.01)
	> 250–500 m	469	1.04	(0.94–1.15)	1.02	(0.91–1.14)
	> 500 m	2′732	1.00		1.00	
Leukemia	0–50 m	3	1.08	(0.33–3.54)	1.06	(0.32–3.51)
	> 50–100 m	9	0.77	(0.39–1.53)	0.76	(0.39–1.52)
	> 100–250 m	59	1.04	(0.79–1.36)	1.02	(0.77–1.36)
	> 250–500 m	141	1.04	(0.86–1.25)	1.02	(0.84–1.24)
	> 500 m	823	1.00		1.00	
Leukemia, 0–5 y	0–50 m	1	0.58	(0.08–4.39)	0.60	(0.08–4.50)
	> 50–100 m	6	0.84	(0.36–1.94)	0.85	(0.37–1.98)
	> 100–250 m	29	0.98	(0.66–1.45)	0.97	(0.65–1.46)
	> 250–500 m	68	0.98	(0.75–1.28)	0.97	(0.73–1.29)
	> 500 m	412	1.00		1.00	
CNS tumors	0–50 m	3	1.46	(0.43–4.91)	1.37	(0.40–4.67)
	> 50–100 m	6	0.88	(0.38–2.03)	0.87	(0.37–2.03)
	> 100–250 m	46	1.02	(0.75–1.40)	1.03	(0.75–1.43)
	> 250–500 m	84	0.82	(0.65–1.04)	0.83	(0.64–1.06)
	> 500 m	631	1.00		1.00	

^a^Odds ratio of unadjusted conditional logistic regression model

^b^95% confidence interval

^c^Odds ratio of conditional logistic regression model adjusted for ambient level of NO_2_, distance to the nearest highway, socio-economic position of the immediate neighborhood area, degree of urbanization of the municipality of residence, and years of existence of a general cantonal cancer registry

### Meta-analysis of childhood leukemia

**Fig. 2:**
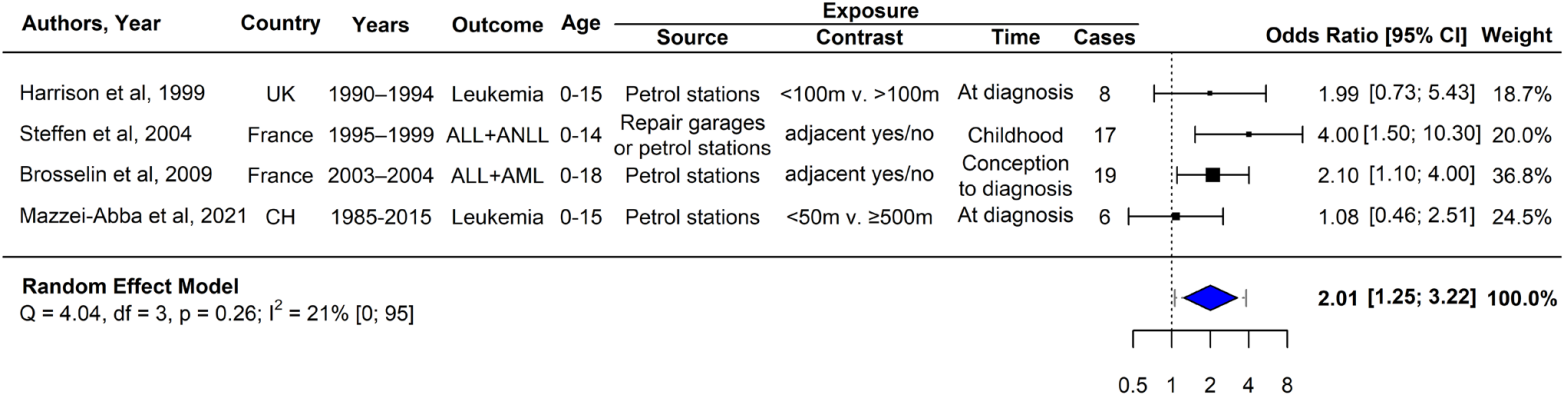
Forest plot of a meta-analysis pooling results of the current analysis with those of three original studies of the residential proximity to petrol stations and risk of childhood leukemia. *ALL* acute lymphocytic leukemia; *AML* acute myeloid leukemia; *ANLL* acute non-lymphocytic leukemia; *CI* confidence interval; *UK* United Kingdom; *CH* Switzerland

The summary relative risk estimate from the random-effects model of our meta-analysis including the results of the three previous studies and our own analysis on the risk of childhood leukemia was 2.01 (1.25–3.22). All four original studies reported positive associations with odds ratios ranging from 1.08 to 4.00 (Fig. 2). In spite of varying definitions and periods of exposure, estimated heterogeneity in our model was low (*I*^2^ = 21%). However, given the small number of included studies, the 95% confidence interval of the heterogeneity statistic was large (0–95%).

### Exposure at birth

The analysis of residence at birth included 16 children with cancer living in close proximity to a petrol station, three with leukemia and three with CNS tumors (Table 4). We found little indication of an association between proximity to petrol stations and risk of childhood cancer over the whole study period. The adjusted odds ratio of a cancer diagnosis for children living close to a petrol station compared with unexposed children (> 500 m) was 0.95 (0.57–1.59) for all cancers combined, 0.52 (0.16–1.67) for leukemia, 0.91 (0.28–3.00) for leukemia in children under 5 years of age, and 1.11 (0.33–3.68) for CNS tumors (Table 4). Effect estimates for any cancer were again higher when we restricted to more recent time periods. The adjusted odds ratio of any cancer diagnosis was 1.29 (0.70–2.35) during 1995–2015 (Table S2) and 1.70 (0.89–3.24) during 2000–2015 (Table S3).

**Table 4:** Associations between childhood cancer and proximity to petrol stations at birth

Outcome	Distance	Cases	OR^a^	95% CI^b^	Adj OR^c^	95% CI^b^
All cancers	0–50 m	16	0.91	(0.55–1.53)	0.95	(0.57–1.59)
	> 50–100 m	36	0.75	(0.54–1.06)	0.79	(0.56–1.11)
	> 100–250 m	234	0.84	(0.73–0.96)	0.87	(0.76–1.01)
	> 250–500 m	611	0.96	(0.88–1.05)	0.99	(0.90–1.09)
	> 500 m	4′024	1.00		1.00	
Leukemia	0–50 m	3	0.52	(0.16–1.67)	0.52	(0.16–1.67)
	> 50–100 m	13	0.87	(0.49–1.54)	0.90	(0.51–1.60)
	> 100–250 m	78	0.90	(0.71–1.15)	0.92	(0.72–1.17)
	> 250–500 m	194	0.99	(0.84–1.15)	1.00	(0.84–1.18)
	> 500 m	1′254	1.00		1.00	
Leukemia, 0–5 y	0–50 m	3	0.94	(0.29–3.09)	0.91	(0.28–3.00)
	> 50–100 m	8	0.84	(0.41–1.74)	0.84	(0.41–1.75)
	> 100–250 m	41	0.80	(0.58–1.11)	0.79	(0.56–1.10)
	> 250–500 m	108	0.92	(0.75–1.14)	0.91	(0.73–1.14)
	> 500 m	711	1.00		1.00	
CNS tumors	0–50 m	3	1.04	(0.31–3.43)	1.11	(0.33–3.68)
	> 50–100 m	6	0.58	(0.26–1.33)	0.65	(0.28–1.49)
	> 100–250 m	50	0.80	(0.59–1.07)	0.88	(0.65–1.20)
	> 250–500 m	121	0.87	(0.71–1.06)	0.94	(0.76–1.15)
	> 500 m	866	1.00		1.00	

^a^Odds ratio of unadjusted conditional logistic regression model

^b^95% confidence interval

^c^Odds ratio of conditional logistic regression model adjusted for ambient level of NO_2_, distance to the nearest highway, socio-economic position of the immediate neighborhood area, degree of urbanization of the municipality of residence, and years of existence of a general cantonal cancer registry

## Discussion

### Main findings

This registry-based case–control study is suggestive of an increased risk of childhood cancer (all diagnostic groups combined) among children living in close proximity of a petrol station when assessing exposure at diagnosis. The observed association was stronger during the last two decades compared to the full study period (1985–2015), and strongest during 2000–2015, a sub-period, during which ambient exposure levels were substantially lower. The observed association appeared to be stronger for CNS tumors; there was little indication of an association for leukemia nor for leukemia in children under 5 years of age. However, a meta-analysis pooling our results with those from three previous studies found evidence of an increased risk of childhood leukemia. In the analysis of residence at birth, we found little indication of an association between proximity to petrol stations and childhood cancer for the entire study period. However, we did observe some indication of an increase in cancer risk during the later sub-periods.

### Results in the context of previous studies

Previous studies investigating cancer risks associated with residential proximity to petrol stations focused exclusively on childhood leukemia. Using a fixed effects model, a first meta-analysis of three previous studies, which included two French studies (Brosselin et al. 2009; Steffen et al. 2004) and an English study (Harrison et al. 1999), reported a summary relative risk of 1.59 (95% CI 0.70–3.62) (Carlos-Wallace et al. 2016). The authors subsequently updated their meta-analysis in response to a commentary (Infante 2017); this suggested using total childhood leukemia instead of AML as outcome and distance to petrol stations instead of automotive repair garages as exposure metric because the latter have fewer petrol pumps and exposure to gasoline vapors in their vicinity should therefore be lower. In their updated meta-analysis, the authors obtained a higher estimate of the summary relative risk of 2.42 (1.51–3.89) (Steinmaus and Smith 2017).

Our results for leukemia individually differed from the findings of these meta-analyses and the included original studies that all reported odds ratios of 1.5 or above (Brosselin et al. 2009; Harrison et al. 1999; Steffen et al. 2004). However, when pooling the results of these studies with our own, the estimated summary relative risk was reduced compared to the second meta-analysis (Steinmaus and Smith 2017), but still showed evidence of an increased risk, with all four included studies showing positive associations. A possible explanation of these differences between original studies is the smaller number of exposed cases included in our analysis (6 vs. 8–19 in the other studies) which limited power and the precision of our effect estimates. The studies also differed regarding exposure definitions and assessment: while we classified children living within 50 m of a petrol station in the highest exposure category, the English study (Harrison et al. 1999) used a 100 m threshold and the two French studies considered only children living directly adjacent to a petrol station as exposed (Brosselin et al. 2009; Steffen et al. 2004). In our analyses, we considered only one discrete point in time (either date of diagnosis or birth) whereas the French studies accounted for exposure at any time between conception and diagnosis, which might have resulted in some misclassifications of our cases. Both French studies assessed exposure through interviews with the children’s mothers and were thus potentially affected by recall bias. Both the English (Harrison et al. 1999) and our study obtained geocoded address information on children’s residences and location of petrol stations from routine data sets and used Geographic Information System software to calculate distances between them. Finally, the studies also differed with regard to the confounders included in the analyses. Apart from one French study (Brosselin et al. 2009), which adjusted for level of urbanization, none of the three original studies included a measure of background levels of air pollution. The English study (Harrison et al. 1999) ran a separate analysis for distance to high traffic roads as exposure metric, finding a positive association, but did not include it to adjust the analyses of the impact of petrol stations.

To our knowledge, no previous study assessed the risks of childhood CNS tumors in relation to proximity of residence to petrol stations. However, several studies have reported increased risks for CNS tumors in the offspring of parents with occupational exposures to benzene (Ali et al. 2004; Cordier et al. 1997; Cordier et al. 2001; Keegan et al. 2013; McKean-Cowdin et al. 1998; Peters et al. 2014; Peters et al. 2013) and recently studies have reported evidence of increased risk of subtypes of CNS tumors from exposure to ambient levels of benzene (Danysh et al. 2015; Raaschou-Nielsen et al. 2018; von Ehrenstein et al. 2016). These studies reported positive associations between outdoor air levels of benzene at place of residence and risk of childhood brain tumors, particularly primitive neuroectodermal tumors (PNET) (Danysh et al. 2015; von Ehrenstein et al. 2016). In our study, the low number of CNS cases precluded any analysis of specific subtypes. The only other study looking at all CNS tumors combined, contrary to our results, found no association with ambient levels of benzene (Danysh et al. 2015).

### Strengths and weaknesses

A major strength of our study is that the risk of selection bias or differential misclassification were minimized. Cases were identified from a population-based cancer registry with coverage above 95% for most of the study period and controls were sampled from national censuses and can thus be considered representative of the population at risk. Exposure assessment did not require any active participation by study members. The residential locations of cases were obtained from the SCCR, which routinely verifies reported addresses at diagnosis and reconstructs address histories back to birth by contacting municipal population registries. Moreover, we assessed exposure based on exact geocodes of children’s place of residence (< 50 m precise for 93% of cases) and of the locations of petrol stations, minimizing the risk of misclassification bias. Among the 24 exposed cases in our full analysis at diagnosis, the margin of error of the geocodes of the residential address was less than 100 m in 22 instances; it was less than 50 m for all 17 exposed cases included in the analysis for the 2000–2015 sub-period. Similarly, the geocodes were accurate to < 100 m for 13 out of the 16 cases exposed at birth and accurate to < 50 m for all 11 exposed cases included in the analysis for the 2000–2015 sub-period. A Greek study suggested that benzene concentration in outdoor air reverts to background levels already at 50 m from a petrol station (Karakitsios et al. 2007). Furthermore, we could adjust our analyses for background levels of air pollution and proximity to highways, two potentially important confounders (Filippini et al. 2019; von Ehrenstein et al. 2016).

The main limitation of our study was the small number of exposed cases that resulted in wide confidence intervals of our effect estimates. These small numbers of exposed cases also precluded separate analyses of other main diagnostic groups such as lymphoma or subtypes of leukemia and CNS tumors. Furthermore, geocodes of petrol stations were available only from 1995 onwards, and only intermittently until 2011, so we calculated the distance to the nearest petrol station based on the latest preceding business survey. This is likely to have led to some misclassification error, particularly during the first decade of the study period; however, we would expect this misclassification to be non-differential and thus to have biased the results to the null. The fact that our effect estimates increased in the analyses of the more recent time periods when the risk of exposure misclassification was minimized, supports this interpretation. Moreover, our analyses focused on exposure at time of birth or diagnosis and could not take into account entire residential histories including address during gestation. This is likely to have led to some degree of exposure misclassification. Finally, in an observational study we cannot rule out residual confounding by unobserved covariates. Crucially, we had no data on reduced benzene emissions from petrol stations due to the fitting of vapor recovery nozzles or other technical installations, which have helped reduce evaporative loss substantially in recent decades.

### Interpretation

In contrast to previous studies and meta-analyses, our study individually found little indication of increased leukemia risks among children living close to petrol stations. This was unexpected given the known link in occupationally exposed adults and leukemia, and the existing evidence of associations of childhood leukemia with ambient (Filippini et al. 2019) and parental occupational exposure (Carlos-Wallace et al. 2016). However, the confidence interval of our effect estimate for leukemia, which were large due to the small number of exposed cases, are still compatible with considerably increased risks in close vicinity of petrol stations and, in particular, also with results from the meta-analyses of previous studies. This interpretation was also corroborated by a meta-analysis pooling our results with those of previous studies showing evidence of an increased risk of childhood leukemia.

When grouping all cancer types together, our study did find weak evidence of a positive association between proximity of place of residence to petrol stations and risk of childhood cancer, particularly during the second half of the study period. Assuming a causal effect, the observed association may have become stronger over time for two reasons. First, non-differential exposure misclassification may have biased results to the null during the earlier years of the study period. Indeed, for lack of any earlier business survey data, we were obliged to back-extrapolate the locations of petrol station from the 1995 survey for the first 10 years from 1985 to 1994. Given the growth rate of petrol stations observed over the subsequent years, the misclassification rate was almost certainly higher during the first half of the study period compared to the second. Also, geocodes of children’s place of residence became more accurate over the study period. For cases diagnosed between 1985 and 2010, residential geocodes of controls were obtained from the decennial censuses closest to a case’s year of diagnosis, whereas from 2010 onwards annual censuses and updates of residential addresses were available. Secondly, background levels of ambient benzene concentration decreased substantially over the study period. This was likely the result of the lower benzene content in petrol due to strict regulation enacted as of 2000 and the simultaneous surge in popularity of diesel powered vehicles in Switzerland, the proportion of which among all cars grew from 4.0% in 2000 to 29.6% in 2020 according to the Swiss Federal Statistical Office. This combined effect had also been observable in France (Vardoulakis et al. 2005). As petrol stations are often strategically located on heavy trafficked roads, it is possible that exposure levels near petrol stations declined at a slower rate such that the exposure contrasts between those children residing close to petrol stations and those further away increased over time.

We did find some indication of an increased risk for CNS tumors among children living in close proximity of petrol stations. This was more exploratory in nature and based on only five exposed cases. No other study has investigated the risk of childhood CNS tumors in relation to this particular exposure, and the evidence of associations with traffic-related air pollution and ambient air concentrations of benzene is not conclusive. It is well possible that the apparent association observed in our study for CNS tumors is a result of sampling variability.

Finally, considering that the six cases of leukemia and five CNS tumors could not account for the overall excess of cancer cases observed in our analysis at diagnosis, in an ex post analysis we tallied the frequency of the main ICCC3 diagnostic groups among all 24 exposed cancer cases. We counted three cases of neuroblastomas, which represented the biggest relative excess (based on proportions among all cases) and the third most important diagnostic group after leukemias and CNS tumors in absolute numbers. This finding concurs with a Californian study that also observed a higher incidence of neuroblastomas among the offspring of women exposed to higher ambient levels of benzene around their homes during pregnancy (Heck et al. 2013).

## Conclusion

Our study provides weak support for an increased risk of all childhood cancers among children living in the immediate proximity (≤ 50 m) of petrol stations. The number of exposed cases included in our study was small and does not allow any conclusions for specific diagnostic groups. However, a meta-analysis combining results of the present and three previous studies suggests that living near a petrol station is associated with an increased risk of childhood leukemia.

## Supplementary Information

Below is the link to the electronic supplementary material.

Supplementary file1 (DOCX 61 KB)

## Data Availability

The Swiss Childhood Cancer Registry is the permanent repository of data on childhood cancer cases used in this study. This data must not be made publicly available for both legal and ethical reasons as this would compromise patient confidentiality and participant privacy. Interested researchers may contact the corresponding author or the Swiss Childhood Cancer Registry (http://childhoodcancerregistry.ch/) via its online contact form for further information.

## Code availability

The code will be available from the authors upon request.

## References

[CR1] Ali R (2004). A case-control study of parental occupation, leukemia, and brain tumors in an industrial city in Taiwan. J Occup Environ Med.

[CR2] Bayerisches Landesamt für Umwelt (2013). Benzol UmweltWissen—Schadstoffe.

[CR3] Brosselin P (2009). Acute childhood leukaemia and residence next to petrol stations and automotive repair garages: the ESCALE study (SFCE). Occup Environ Med.

[CR4] Carlos-Wallace FM, Zhang L, Smith MT, Rader G, Steinmaus C (2016). Parental, in utero, and early-life exposure to benzene and the risk of childhood leukemia: a meta-analysis. Am J Epidemiol.

[CR5] Cordier S (1997). Parental occupation, occupational exposure to solvents and polycyclic aromatic hydrocarbons and risk of childhood brain tumors (Italy, France, Spain). Cancer Causes Control.

[CR6] Cordier S (2001). Parental occupations and childhood brain tumors: results of an international case-control study. Cancer Causes Control.

[CR7] Danysh HE, Mitchell LE, Zhang K, Scheurer ME, Lupo PJ (2015). Traffic-related air pollution and the incidence of childhood central nervous system tumors: Texas, 2001–2009. Pediatr Blood Cancer.

[CR8] Filippini T (2019). Association between outdoor air pollution and childhood leukemia: a systematic review and dose-response meta-analysis. Environ Health Perspect.

[CR9] Harrison RM, Leung PL, Somervaille L, Smith R, Gilman E (1999). Analysis of incidence of childhood cancer in the West Midlands of the United Kingdom in relation to proximity to main roads and petrol stations. Occup Environ Med.

[CR10] Heck JE, Park AS, Qiu J, Cockburn M, Ritz B (2013). An exploratory study of ambient air toxics exposure in pregnancy and the risk of neuroblastoma in offspring. Environ Res.

[CR11] Heldstab J, Leippert F, Wüthrich P, Künzle T (2011). NO2 ambient concentrations in Switzerland—Modelling results for 2005, 2010, 2015.

[CR12] IARC (2017). IARC monographs on the evaluation of carcinogenic risks to humans.

[CR13] Infante PF (2017). Residential proximity to gasoline stations and risk of childhood leukemia. Am J Epidemiol.

[CR14] Karakitsios SP, Delis VK, Kassomenos PA, Pilidis GA (2007). Contribution to ambient benzene concentrations in the vicinity of petrol stations: Estimation of the associated health risk. Atmos Environ.

[CR15] Keegan TJ (2013). Case-control study of paternal occupation and social class with risk of childhood central nervous system tumours in Great Britain, 1962–2006. Br J Cancer.

[CR16] Konstantinoudis G (2017). Spatial clustering of childhood leukaemia in Switzerland: a nationwide study. Int J Cancer.

[CR17] Konstantinoudis G (2020). Bayesian spatial modelling of childhood cancer incidence in Switzerland using exact point data: a nationwide study during 1985–2015. Int J Health Geogr.

[CR18] McKean-Cowdin R, Preston-Martin S, Pogoda JM, Holly EA, Mueller BA, Davis RL (1998). Parental occupation and childhood brain tumors: astroglial and primitive neuroectodermal tumors. J Occup Environ Med.

[CR19] Morales Terrés IM, Miñarro MD, Ferradas EG, Caracena AB, Rico JB (2010). Assessing the impact of petrol stations on their immediate surroundings. J Environ Manage.

[CR20] Panczak R (2012). A Swiss neighbourhood index of socioeconomic position: development and association with mortality. J Epidemiol Community Health.

[CR21] Peters S (2013). Parental occupational exposure to engine exhausts and childhood brain tumors. Int J Cancer.

[CR22] Peters S (2014). Childhood brain tumours: associations with parental occupational exposure to solvents. Br J Cancer.

[CR23] R Core Team (2019). R: A language and environment for statistical computing.

[CR24] Raaschou-Nielsen O, Hvidtfeldt UA, Roswall N, Hertel O, Poulsen AH, Sørensen M (2018). Ambient benzene at the residence and risk for subtypes of childhood leukemia, lymphoma and CNS tumor. Int J Cancer.

[CR25] SAEFL (2004). Modelling of NO2 and benzene ambient concentrations in Switzerland 2000 to 2010 Environmental Documentation.

[CR26] Schindler M (2015). Death certificate notifications in the Swiss Childhood Cancer Registry: assessing completeness and registration procedures. Swiss Med Wkly.

[CR27] Steffen C (2004). Acute childhood leukaemia and environmental exposure to potential sources of benzene and other hydrocarbons; a case-control study. Occup Environ Med.

[CR28] Steinmaus C, Smith MT (2017). Steinmaus and smith respond to “Proximity to gasoline stations and childhood leukemia”. Am J Epidemiol.

[CR29] Steliarova-Foucher E (2017). International incidence of childhood cancer, 2001–10: a population-based registry study. Lancet Oncol.

[CR30] Steliarova-Foucher E, Stiller C, Lacour B, Kaatsch P (2005). International classification of childhood cancer, third edition. Cancer.

[CR31] Taylor JMG (1986). Choosing the number of controls in a matched case-control study, some sample size, power and efficiency considerations. Stat Med.

[CR32] Vardoulakis S, Gonzalez-Flesca N, Fisher BEA, Pericleous K (2005). Spatial variability of air pollution in the vicinity of a permanent monitoring station in central Paris. Atmos Environ.

[CR33] von Ehrenstein OS, Heck JE, Park AS, Cockburn M, Escobedo L, Ritz B (2016). In utero and early-life exposure to ambient air toxics and childhood brain tumors: a population-based case-control study in California, USA. Environ Health Perspect.

[CR34] Wilbur S et al. (2007) Toxicological profile for benzene. US Department of Health and Human Services, Agency for Toxic Substances and Disease Registry, Atlanta, GA 37184171

