# Supplementary material for: Childhood cancer and residential proximity to petrol stations: a nationwide registry-based case–control study in Switzerland and an updated meta-analysis
Source: Int Arch Occup Environ Health. 2021 Oct 15;95(5):927–38. doi: 10.1007/s00420-021-01767-y (PMC9203398; doi:10.1007/s00420-021-01767-y)

**Supplementary Material**

**Childhood cancer and residential proximity to petrol stations: a nationwide registry-based case-control study in Switzerland and an updated meta-analysis**

**Antonella Mazzei^1^, Garyfallos Konstantinoudis^1,2^, Christian Kreis^1^, Manuel Diezi^3^, Roland A Ammann^4^, Marcel Zwahlen^1^, Claudia Kühni^1^, Ben D. Spycher^1^**

1. Institute of Social and Preventive Medicine, University of Bern, Bern, Switzerland
2. MRC Centre for Environment and Health, Department of Epidemiology and Biostatistics, School of Public Health, Imperial College London, London, UK
3. Centre hospitalier universitaire Vaudois, Lausanne University Hospital, Lausanne, Switzerland
4. Division of Pediatric Hematology/Oncology, Department of Pediatrics Inselspital, Bern University Hospital, Bern, Switzerland

**Corresponding author:**

Ben D Spycher

Institute of Social and Preventive Medicine (ISPM), University of Bern, Mittelstrasse 43, 3012 Bern, Switzerland.

Tel: +41 31 631 33 46

Fax: +41 31 631 35 20

E-mail: [ben.spycher@ispm.unibe.ch](mailto:ben.spycher@ispm.unibe.ch)

# Tables

**Table S1.** Associations between childhood cancer and proximity to petrol stations at diagnosis, 1995-2015

| **Outcome** | **Distance** | **Cases** | **OR^a^** | **95% CI^b^** | **Adj OR^c^** | **95% CI^b^** |
| --- | --- | --- | --- | --- | --- | --- |
| All cancers | 0-50 m | 18 | 1.40 | (0.85-2.29) | 1.41 | (0.86-2.32) |
|  | >50-100 m | 41 | 1.06 | (0.77-1.46) | 1.08 | (0.78-1.50) |
|  | >100-250 m | 215 | 0.90 | (0.78-1.04) | 0.93 | (0.80-1.08) |
|  | >250-500 m | 599 | 1.09 | (1.00-1.20) | 1.12 | (1.02-1.23) |
|  | >500 m | 3’500 | 1.00 |  | 1.00 |  |
| Leukemia | 0-50 m | 3 | 0.69 | (0.21-2.22) | 0.70 | (0.22-2.27) |
|  | >50-100 m | 14 | 1.06 | (0.61-1.85) | 1.10 | (0.63-1.92) |
|  | >100-250 m | 74 | 1.04 | (0.81-1.33) | 1.07 | (0.83-1.38) |
|  | >250-500 m | 174 | 1.06 | (0.89-1.25) | 1.08 | (0.90-1.28) |
|  | >500 m | 1’054 | 1.00 |  | 1.00 |  |
| Leukemia, 0-5y | 0-50 m | 1 | 0.36 | (0.05-2.63) | 0.37 | (0.05-2.74) |
|  | >50-100 m | 9 | 1.13 | (0.56-2.26) | 1.19 | (0.59-2.40) |
|  | >100-250 m | 36 | 0.97 | (0.68-1.38) | 1.00 | (0.70-1.44) |
|  | >250-500 m | 86 | 1.03 | (0.81-1.31) | 1.08 | (0.84-1.39) |
|  | >500 m | 526 | 1.00 |  | 1.00 |  |
| CNS tumors | 0-50 m | 4 | 1.63 | (0.57-4.71) | 1.60 | (0.55-4.64) |
|  | >50-100 m | 9 | 1.09 | (0.55-2.19) | 1.16 | (0.58-2.33) |
|  | >100-250 m | 51 | 0.93 | (0.70-1.26) | 1.00 | (0.73-1.35) |
|  | >250-500 m | 111 | 0.90 | (0.73-1.11) | 0.95 | (0.76-1.18) |
|  | >500 m | 793 | 1.00 |  | 1.00 |  |

^a^Odds ratio of unadjusted conditional logistic regression model; ^b^95% confidence interval; ^c^Odds ratio of conditional logistic regression model adjusted for ambient level of NO2, distance to the nearest highway, socio-economic position of the immediate neighborhood area, degree of urbanization of the municipality of residence, and years of existence of a general cantonal cancer registry.

**Table S2.** Associations between childhood cancer and proximity to petrol stations at birth, 1995-2015

| **Outcome** | **Distance** | **Cases** | **OR^a^** | **95% CI^b^** | **Adj OR^c^** | **95% CI^b^** |
| --- | --- | --- | --- | --- | --- | --- |
| All cancers | 0-50 m | 12 | 1.15 | (0.63-2.09) | 1.29 | (0.70-2.35) |
|  | >50-100 m | 21 | 0.68 | (0.44-1.07) | 0.78 | (0.50-1.21) |
|  | >100-250 m | 142 | 0.77 | (0.65-0.92) | 0.87 | (0.72-1.04) |
|  | >250-500 m | 372 | 0.88 | (0.79-0.99) | 0.98 | (0.87-1.10) |
|  | >500 m | 2’338 | 1.00 |  | 1.00 |  |
| Leukemia | 0-50 m | 2 | 0.54 | (0.13-2.23) | 0.63 | (0.15-2.65) |
|  | >50-100 m | 9 | 0.85 | (0.43-1.70) | 0.95 | (0.47-1.90) |
|  | >100-250 m | 45 | 0.75 | (0.55-1.03) | 0.82 | (0.60-1.13) |
|  | >250-500 m | 119 | 0.88 | (0.72-1.07) | 0.96 | (0.77-1.18) |
|  | >500 m | 755 | 1.00 |  | 1.00 |  |
| Leukemia, 0-5y | 0-50 m | 2 | 0.83 | (0.20-3.52) | 0.93 | (0.22-3.96) |
|  | >50-100 m | 7 | 0.94 | (0.43-2.07) | 1.02 | (0.46-2.25) |
|  | >100-250 m | 29 | 0.76 | (0.52-1.12) | 0.81 | (0.54-1.20) |
|  | >250-500 m | 69 | 0.80 | (0.62-1.04) | 0.86 | (0.65-1.14) |
|  | >500 m | 470 | 1.00 |  | 1.00 |  |
| CNS tumors | 0-50 m | 2 | 1.60 | (0.35-7.29) | 1.74 | (0.38-7.95) |
|  | >50-100 m | 3 | 0.51 | (0.16-1.62) | 0.61 | (0.19-1.97) |
|  | >100-250 m | 37 | 0.90 | (0.64-1.28) | 1.10 | (0.76-1.58) |
|  | >250-500 m | 70 | 0.77 | (0.59-0.99) | 0.89 | (0.68-1.17) |
|  | >500 m | 516 | 1.00 |  | 1.00 |  |

^a^Odds ratio of unadjusted conditional logistic regression model; ^b^95% confidence interval; ^c^Odds ratio of conditional logistic regression model adjusted for ambient level of NO2, distance to the nearest highway, socio-economic position of the immediate neighborhood area, degree of urbanization of the municipality of residence, and years of existence of a general cantonal cancer registry.

**Table S3.** Associations between childhood cancer and proximity to petrol stations at birth, 2000-2015

| **Outcome** | **Distance** | **Cases** | **OR^a^** | **95% CI^b^** | **Adj OR^c^** | **95% CI^b^** |
| --- | --- | --- | --- | --- | --- | --- |
| All cancers | 0-50 m | 11 | 1.63 | (0.86-3.10) | 1.70 | (0.89-3.24) |
|  | >50-100 m | 13 | 0.57 | (0.32-0.99) | 0.60 | (0.34-1.06) |
|  | >100-250 m | 100 | 0.78 | (0.64-0.97) | 0.83 | (0.67-1.03) |
|  | >250-500 m | 245 | 0.85 | (0.74-0.98) | 0.89 | (0.77-1.03) |
|  | >500 m | 1’486 | 1.00 |  | 1.00 |  |
| Leukemia | 0-50 m | 2 | 0.86 | (0.20-3.68) | 0.94 | (0.22-4.04) |
|  | >50-100 m | 5 | 0.58 | (0.23-1.44) | 0.60 | (0.24-1.51) |
|  | >100-250 m | 33 | 0.76 | (0.53-1.10) | 0.79 | (0.54-1.14) |
|  | >250-500 m | 79 | 0.81 | (0.63-1.03) | 0.84 | (0.65-1.09) |
|  | >500 m | 521 | 1.00 |  | 1.00 |  |
| Leukemia, 0-5y | 0-50 m | 2 | 1.11 | (0.26-4.83) | 1.23 | (0.28-5.38) |
|  | >50-100 m | 5 | 0.82 | (0.33-2.06) | 0.87 | (0.34-2.20) |
|  | >100-250 m | 24 | 0.79 | (0.52-1.21) | 0.84 | (0.54-1.31) |
|  | >250-500 m | 52 | 0.76 | (0.56-1.03) | 0.83 | (0.60-1.13) |
|  | >500 m | 354 | 1.00 |  | 1.00 |  |
| CNS tumors | 0-50 m | 2 | 2.19 | (0.46-10.43) | 2.13 | (0.44-10.24) |
|  | >50-100 m | 1 | 0.24 | (0.03-1.75) | 0.27 | (0.04-1.99) |
|  | >100-250 m | 28 | 1.03 | (0.68-1.55) | 1.15 | (0.75-1.76) |
|  | >250-500 m | 49 | 0.84 | (0.61-1.15) | 0.90 | (0.65-1.26) |
|  | >500 m | 314 | 1.00 |  | 1.00 |  |

^a^Odds ratio of unadjusted conditional logistic regression model; ^b^95% confidence interval; ^c^Odds ratio of conditional logistic regression model adjusted for ambient level of NO2, distance to the nearest highway, socio-economic position of the immediate neighborhood area, degree of urbanization of the municipality of residence, and years of existence of a general cantonal cancer registry.

# Figures

**Figure S1.** Number of petrol stations in Switzerland 1995-2015


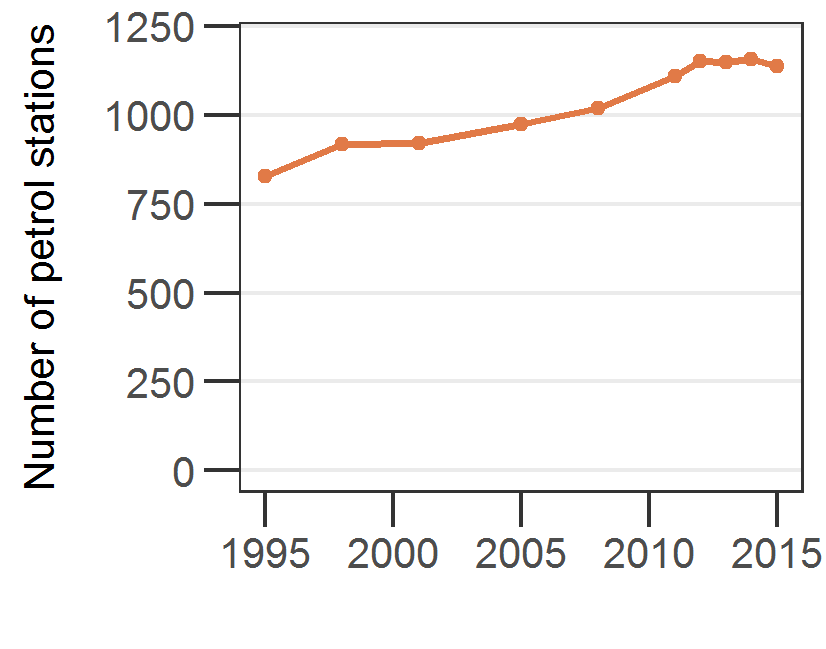

Supplement: Supplementary file 1 — Supplementary file1 (DOCX 61 KB) [file 420_2021_1767_MOESM1_ESM.docx]
